# Dietary modulation of the gut microbiome as a supportive strategy in the treatment of amyotrophic lateral sclerosis – a narrative review

**DOI:** 10.1007/s43440-025-00800-y

**Published:** 2025-11-04

**Authors:** Aneta Kiecka, Marian Szczepanik

**Affiliations:** Faculty of Health Sciences, Institute of Physiotherapy, Chair of Biomedical Sciences, Jagiellonian University Medical College, Kopernika 7a, Kraków, 31-034 Poland

**Keywords:** Amyotrophic lateral sclerosis, Gut microbiota-brain axis, Microbiota, Probiotics, Diets

## Abstract

Amyotrophic lateral sclerosis (ALS) is a fatal neurodegenerative disease leading to permanent damage to the central and peripheral motor neurons. Currently, there is no effective treatment for ALS, and therapy focuses solely on slowing the progression of the disease. Recent studies show that gut microbiota plays an important role in the development of neurodegenerative diseases. Altered gut microbiota has also been found in ALS. These changes have prompted the search for alternative forms of ALS treatment, focusing on changing the microbial composition of the gut. It has been noted that diet, probiotics, prebiotics and vitamins can all influence the course of ALS. Another interesting issue is fecal microbiota transplantation, which is already used in the treatment of certain intestinal diseases and could potentially be useful in the treatment of ALS. This review summarizes current knowledge on the impact of gut microbiota on the neurodegenerative process in ALS, with particular emphasis on the role of diet and probiotics. It also discusses potential mechanisms and highlights future research directions in this emerging field.

## Introduction

Amyotrophic lateral sclerosis (ALS) is a fatal neurodegenerative disease characterized by progressive damage to upper and lower motor neurons in the brain and spinal cord, which leads to muscle weakness, paralysis, and death from respiratory failure within five years of symptom onset [1]. ALS symptoms initially develop in the limb muscles or bulbar muscles, then spread to other parts of the body, eventually affecting the respiratory muscles. The primary symptoms of ALS include muscle spasm, stiffness, dysarthria, dysphagia, emotional instability, and drooling [1]. The incidence of this disease is 0.6 to 3.8 per 100,000 persons per year [2]. It has been observed that about 10% of ALS cases are familial (fALS), while 90% are sporadic (sALS), but in the clinical picture, they are indistinguishable. In risk factors for ALS include age over 55, male gender, stress, exposure to heavy metals, and environmental pollution. However, the full mechanism of disease, etiology, and risk factors is not fully understood [3]. Currently, there is no effective treatment for ALS, and treatment focuses on slowing the disease’s progression. The most commonly used drugs approved by the FDA for oral use are riluzole and edaravone. These drugs inhibit the effects of oxidative stress by eliminating oxidized lipids and hydroxyl radicals [4]. Another therapy involves the administration of sodium phenylbutyrate [4]. Apart from pharmacotherapy, the available new therapeutic methods include gene therapy and the use of stem cells [5]. Despite significant progress, new treatments for ALS are constantly being sought. Current studies show that gut microbiota may influence the development of ALS by modulating the immune response [6]. Abnormal gut microbiota may contribute to the exacerbation of inflammation, which is crucial in the pathogenesis of ALS. Disruption within gut microbiota composition may influence the excessive activation of microglia, which in turn leads to neuroinflammatory reactions that accelerate neurodegeneration and disease progression [6]. In turn, a reduction in the number of anti-inflammatory bacteria, such as *Faecalibacterium prausnitzii*, in the intestines of ALS patients has been linked to the severity of disease symptoms [7]. Gut dysbiosis can lead to disruption of the intestinal barrier, which can result in the entry of bacterial toxins, such as lipopolysaccharides (LPS), into the bloodstream. The intestinal barrier plays a crucial role in maintaining gut homeostasis by selectively allowing the absorption of nutrients while preventing the translocation of pathogens, toxins, and antigens into the bloodstream. It is composed of a complex structure including epithelial cells joined by tight junctions, a mucus layer, immune cells, and antimicrobial peptides [7, 8]. Disruption of this barrier, commonly referred to as “leaky gut,” can lead to increased intestinal permeability, which has been associated with various inflammatory and autoimmune conditions [8]. Toxins can activate an inflammatory response in the body, including the central nervous system, which may accelerate degenerative processes in ALS [8]. Preliminary studies suggest that therapeutic modulation of the gut microbiota may help restore microbial balance, reduce inflammation, and influence disease progression; however, further research is needed to confirm its potential benefits [8]. This review examines the role of gut microbiota in the pathogenesis and progression of ALS, focusing on how diet, probiotics, and fecal microbiota transplantation may modulate neurodegeneration [8]. Beyond summarizing current evidence, it aims to provide an integrative interpretation of findings and propose novel directions for future studies. A comprehensive and structured literature search was conducted across three major scientific databases: PubMed, Scopus, and Web of Science. The search strategy was designed to identify peer-reviewed articles relevant to the topic of interest, ensuring broad coverage of the available biomedical and multidisciplinary literature.

### The gut microbiota-brain axis and neurodegeneration

**Fig. 1:**
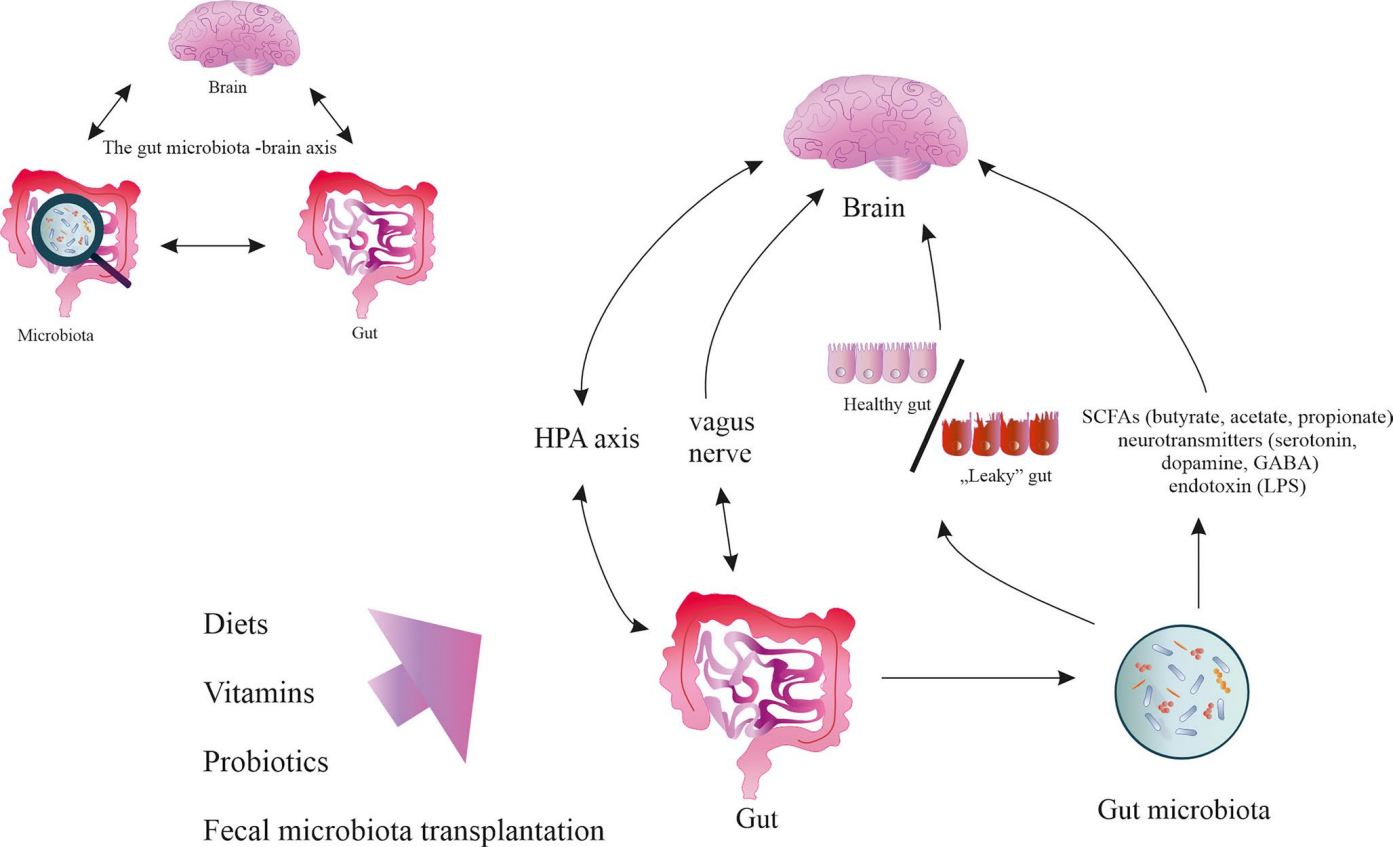
Gut microbiota-brain axis**. ** The gut microbiota–brain axis is a bidirectional communication system between the gut and the brain that includes the nervous, endocrine, and immune systems. The gut microbiota influences brain function, mood, and behavior through the production of various metabolites and signaling molecules, including SCFAs such as acetate, propionate, and butyrate. These SCFAs can cross the blood–brain barrier or act indirectly through immune and neural pathways, modulating inflammation and neurotransmission Additionally, gut microbes are capable of producing and modulating levels of key neurotransmitters such as GABA, serotonin (5-HT), and dopamine. For example, certain Lactobacillus and Bifidobacterium species can produce GABA, while other microbial species influence the synthesis and availability of serotonin by affecting tryptophan metabolism. Through these mechanisms, the gut microbiota plays a crucial role in regulating stress responses, cognitive function, and emotional states

In recent years, the concept of the gut microbiota–brain axis has attracted considerable interest (Fig. 1) [9]. The gut-microbiota axis refers to a complex, bidirectional communication system between the gastrointestinal tract and the central nervous system (CNS), in which intestinal bacteria play a crucial regulatory role. It is estimated that the adult human gut contains over 1,000 species and 7,000 strains of microorganisms, primarily from the *Firmicutes* (e.g., *Lactobacillus*, *Eubacterium*, *Clostridium*) and *Bacteroidetes* (e.g., *Bacteroides*,* Prevotella*) phyla [9, 10]. These microorganisms are involved not only in digestion but also in modulating immune and nervous system functions [11].

Communication between the gut and the brain occurs via several pathways, including the nervous system (particularly the vagus nerve), the hypothalamic–pituitary–adrenal (HPA) axis, and the immune and metabolic systems. The vagus nerve enables bidirectional signal transmission and influences intestinal motility, mucus secretion, cytokine release, and inflammatory as well as neuroendocrine responses [12]. Additionally, gut bacteria synthesize various metabolites and neurotransmitters such as serotonin (5-HT), dopamine, γ-aminobutyric acid (GABA), acetylcholine, and short-chain fatty acids (SCFAs) like butyrate, acetate, and propionate. Notably, approximately 90% of serotonin is produced in the intestines, primarily due to microbial stimulation of enterochromaffin cells [13]. These cells also secrete neuropeptides (e.g., peptide YY, neuropeptide Y, cholecystokinin, GLP-1, GLP-2, substance P) and neurotransmitters (e.g., serotonin, GABA), which may reach the brain and influence its function [14, 15]. Moreover, SCFAs exhibit anti-inflammatory properties, support intestinal barrier integrity, and modulate the activity of glial cells in the CNS [16].

Disruption of gut microbiota homeostasis—referred to as dysbiosis—can result in increased permeability of the intestinal barrier, allowing LPS and bacterial toxins to enter systemic circulation. These molecules may activate Toll-like receptors (TLRs) and trigger immune responses in both the gut and the CNS, contributing to neuroinflammation [17].

Emerging evidence indicates that the gut microbiota–brain axis plays a significant role in the pathogenesis of ALS [18]. In ALS, gut dysbiosis and increased intestinal permeability may contribute to chronic systemic inflammation, which, in turn, promotes disruption of the blood–brain barrier and activation of microglia—key mechanisms implicated in motor neuron degeneration [19]. Although such pathways have been more thoroughly investigated in other neurodegenerative disorders, such as Alzheimer’s and Parkinson’s diseases, similar immune and microbial patterns are increasingly recognized also in ALS. For example, elevated levels of pro-inflammatory cytokines such as interleukin-6 (IL-6) and tumor necrosis factor-α (TNF-α), commonly linked to gut dysbiosis in Alzheimer’s disease, have also been reported in ALS patients and may accelerate disease progression [20, 21]. While the aggregation of α-synuclein is specific for Parkinson’s disease, ALS research suggests similarly that microbial metabolites and dysregulated immune signaling may contribute to abnormal protein aggregation and neurodegeneration [22–25].

Collectively, these findings support the hypothesis that the gut microbiome may influence ALS pathophysiology through immune, metabolic, and neuroinflammatory pathways. Understanding this interaction may offer novel therapeutic strategies targeting the microbiota. The following sections will explore this relationship further in the context of ALS.

### Gut microbiota in ALS

A growing number of studies suggest that gut microbiota dysbiosis may be involved in the pathogenesis and progression of ALS [26, 27]. Although research in this area remains limited, existing data indicate significant differences in gut microbial composition between ALS patients and healthy individuals.

Several clinical studies have reported reduced microbial diversity and notable shifts in dominant bacterial phyla in ALS patients. Fang et al. [28] analyzed stool samples from 25 ALS patients and 25 controls, reporting a decrease in *Firmicutes* and an increase in *Bacteroidetes* in the ALS group, accompanied by reduced microbial diversity as measured by the Shannon index. Similarly, the study by Zeng et al. [29] found decreased *Firmicutes* and *Megamonas*, and increased *Bacteroidetes* in 20 ALS patients compared to healthy subjects. In contrast, another study observed an increase in the *Firmicutes/Bacteroidetes* (F/B) ratio in a smaller cohort, along with higher levels of *Methanobrevibacter* and lower abundance of beneficial genera such as *Faecalibacterium* and *Bacteroides* [30].

While these inconsistencies reflect the heterogeneity of findings across studies, several recurring patterns have emerged. Notably, a decline in SCFA-producing bacteria, particularly *Faecalibacterium prausnitzii*, has been observed in ALS patients [31]. This bacterial species plays a critical role in maintaining intestinal homeostasis due to its anti-inflammatory properties and ability to produce butyrate [31, 32]. A study by Nicholson et al. [33] involving 66 ALS patients, 61 healthy individuals, and 12 patients with other neurodegenerative conditions showed significantly reduced abundance of key butyrate-producing taxa, including *Eubacterium rectale* and *Roseburia intestinalis* (*p* < 0.001).

Other studies have also linked microbiota alterations to immune and metabolic dysfunction in ALS. For example, Hertzberg et al. [34] found a marked reduction of *Prevotella* in ALS patients, along with predicted downregulation of pathways involved in carbohydrate and butyrate metabolism, suggesting possible targets for microbiota-based therapy. Another study that compared ALS patients with two control groups (healthy relatives and matched controls) found significant taxonomic differences, including reduced *Succinivibrionaceae* and *Lachnospiraceae*, and elevated *Streptococcaceae* and *Ruminococcaceae* [35]. LEfSe analysis identified four bacterial families significantly differing between groups (*p* < 0.05; LDA >2.5).

Interestingly, in a study that examined both gut and oral microbiota in ALS subtypes, patients with sALS showed increased fecal F/B ratios, which strongly correlated with microbial translocation into the bloodstream (*r* = 0.8006, *p* < 0.0001) and symptom severity (*r* = 0.9470, *p* < 0.0001) [36]. In contrast, those with bulbar-onset ALS (bALS) displayed a stronger correlation between disrupted oral microbiota and disease severity, suggesting site-specific microbial signatures.

A longitudinal analysis linking altered microbial profiles with plasma lipid metabolites in patients, particularly those involved in fatty acid and acylcarnitine metabolism using Mendelian randomization revealed potential causal relationships between specific lipids and ALS risk [37].

Animal studies have further supported the role of gut microbiota in ALS progression. For example, administration of butyrate to SOD1-G93A transgenic mice restored microbial balance, improved gut barrier function, and prolonged survival [38]. Another study reported microbial heterogeneity among ALS patients and found that changes in SCFA-producing taxa correlated with disease progression, though limited sample sizes and lack of mechanistic data restrict interpretability [39].

A particularly influential study by Blacher et al. [40] demonstrated that certain gut microbes promote nicotinamide production, which in turn supports motor neuron health in SOD1-SOD1-G93A mice, which provided one of the first mechanistic links between gut microbiota and neuroprotection in ALS, highlighting microbial metabolites as potential therapeutic targets.

Despite encouraging associations, current clinical evidence remains insufficient to establish causality. Most human studies are observational, raising the possibility of reverse causation—whether microbiota changes contribute to ALS or result from disease-related factors such as immobility, dietary shifts, or medications. Addressing this challenge requires the use of causal inference methods. Longitudinal sampling, twin studies, and Mendelian randomization approaches may help disentangle the complex interactions between gut dysbiosis and ALS pathophysiology. Rigorous, well-controlled trials are essential to move from correlation to causation and identify clinically actionable targets.

### Dietary components influence the course of ALS by modulating gut microbiota

Diet plays a key role in the development of neurodegenerative diseases, including ALS. A growing number of observational studies indicate that nutrients supplied to the body have a significant impact on the composition and functioning of the gut microbiota, which in turn affects the overall condition of patients and the course of disease [38]. Weight loss, sarcopenia, micronutrient deficiencies, and energy metabolism disorders are commonly observed in people with ALS and correlate with the severity of clinical symptoms and poorer prognosis.

The literature emphasizes that gut microbiota dysbiosis in ALS patients may affect disease pathogenesis by exacerbating inflammation and disrupting the gut–brain axis [37]. Observational and epidemiological data suggest that diets rich in antioxidants, omega-3 fatty acids, and compounds with anti-inflammatory properties, such as polyphenols, may have beneficial effects on neurological function and slow down motor neuron degeneration [39]. In addition, polyphenols may positively affect gut microbiota by promoting the growth of beneficial bacterial strains involved in neurotransmitter synthesis, such as GABA [41]. In human studies, a significant increase in the population of *Bacteroides*, *Bifidobacterium*, and *Lactobacillus* was observed in individuals supplemented with polyphenols [42]. These mechanisms may be particularly relevant in ALS, where neuroinflammation and neuronal dysfunction are key drivers of disease progression [43], and promoting bacteria with anti-inflammatory properties, including *Firmicutes*, *Bacteroidetes*, *Lactobacillus*, *Bifidobacterium*, and SCFA producers such as *Faecalibacterium prausnitzii* and *Roseburia* [44].

Furthermore, bacteria such as *Lactobacillus* and *Bifidobacterium*, commonly present in probiotics, can ferment carbohydrates to produce SCFAs, especially butyrate. Evidence from experimental studies, such as that by Johnson et al. [45], demonstrated that butyrate has anti-inflammatory effects, reducing levels of pro-inflammatory cytokines like TNF-α and IL-6, which are implicated in ALS pathogenesis. It has also been shown that polyphenol metabolites produced by the gut microbiota may reach the brain and modulate its function [46]. Since dysbiosis promotes systemic inflammation, increasing the number of SCFA-producing bacteria may contribute to reducing inflammation, which is an important mechanism in the neurodegeneration observed in ALS [46, 47].

It has been reported that the flavonoid quercetin inhibits the growth of *Enterococcus* and increases *Akkermansia muciniphila*, species implicated in ALS pathophysiology [48]. A major pathological hallmark in ALS results from in vitro and in silico analyses of the effects of myricetin—a natural flavonoid—on the aggregation process of the SOD1 protein, which demonstrated that myricetin binds to interaction regions between SOD1 subunits, stabilizing the native structure and interfering with the formation of amyloid aggregates [49]. Aggregation kinetics using thioflavin confirmed that myricetin reduces SOD1 aggregation in a concentration-dependent manner (IC50 = 15 µM; *p* < 0.001) [49].

While polyphenols and flavonoids show potential for neuroprotection and microbiome modulation, ALS-specific dietary interventions may also require broader nutritional modifications, particularly concerning macronutrient composition. Observational studies suggest a possible association between higher fat intake and better results in ALS treatment. For example, a prospective case-control study found a reduced risk of ALS among meat-eaters (*p* = 0.0006), based on data collected from 1989 to 2002 [50]. Another case-control study conducted in Japan with 153 ALS patients and 306 controls showed that high carbohydrate intake (OR = 2.14, 95% CI 1.05–4.36) and low fat/fatty acid intake were associated with a higher risk of ALS [51]. A Dutch retrospective study reported that high fatty acid intake correlated with a 50–60% risk reduction [52]. However, an American study reported a non-significant trend toward increased ALS risk among individuals on high-fat diets, with those in the highest quartile of fat intake having a 2.7-fold higher risk than those in the lowest (OR = 2.7, 95% CI 1.2–6.1) [53].

While human studies provide correlational evidence linking diet to ALS risk and progression, preclinical studies in animal models offer mechanistic insights into how dietary interventions may influence disease outcomes. Transgenic SOD1-G93A mice, which carry a human mutation responsible for familial ALS, are commonly used to investigate disease mechanisms and potential treatments. In this model, energy-rich and fat-based diets have been shown to improve motor performance and extend survival. For instance, a ketogenic diet improved motor performance by 50% and extended functional duration by 25 days in SOD1-G93A mice [54]. Consistent with these findings, Dupuis et al. [55] demonstrated that a high-calorie diet prolonged survival by 20% (154 vs. 129 days, *p* < 0.001) and enhanced motor coordination on the rotarod test (135 vs. 85 s, *p* < 0.05). Another study [56] reported that a medium-chain triglyceride (MCT)-enriched diet improved mitochondrial oxygen consumption by approximately 35% and enhanced motor performance by 40% in ALS mice, although it did not significantly extend survival (*p* >0.05). These preclinical findings suggest that increased energy availability may mitigate motor neuron degeneration, supporting the rationale for testing high-calorie or fat-based diets in ALS patients.

High-fat diets (HFDs) may also elevate LDL cholesterol levels, which could improve motor neuron survival in the peripheral nervous system. Some human studies have reported an association between elevated cholesterol levels and longer survival in ALS, although this effect weakens when controlling for BMI and nutritional status [57, 58].

Although current evidence highlights the potential of dietary strategies to modulate ALS progression, much of the available data stems from animal studies (Table 1) and observational clinical research (Table 2). Randomized controlled trials in humans are therefore needed to establish causality and determine the therapeutic efficacy of specific dietary interventions.

**Table 1:** Preclinical studies on gut microbiota, diet, and ALS

Model	Intervention / Factor	Dosing and time of treatment	Main findings	References
SOD1-G93A mice	Butyrate supplementation	2% sodium butyrate in drinking water; treatment ~ 2.5 months, started at ~ 63 days of age (pre‑symptomatic).	Restored microbial balance, improved gut barrier, prolonged survival, reduced SOD1 aggregation	[37]
SOD1-G93A mice	*Akkermansia muciniphila* supplementation	Oral gavage of live A. muciniphila into antibiotic‑treated SOD1‑Tg mice; CFU dose not reported in abstract—see original Methods; supplementation period ~ 6–7 weeks.	Improved motor function, increased nicotinamide levels	[40]
SOD1-G93A mice	Ketogenic diet	60% fat / 20% carbohydrate / 20% protein (Research Diets formulation); initiated at day 50 of age; ad libitum.	Improved motor performance (+ 50%), prolonged survival (+ 25 days)	[54]
SOD1-G93A mice	High-calorie diet	High‑energy/high‑fat diet provided ad libitum; exact macronutrient composition not specified in accessible text; see Methods.	20% longer survival, improved motor coordination	[55]
SOD1-G93A mice	MCT-enriched diet	10% (w/w) caprylic triglyceride diet (fat 34%, carbohydrate 46%, protein 20%); started at day 50 of age; ad libitum; motor/survival assessed to ~ 110–135 days.	Improved mitochondrial function and motor performance, no survival benefit	[56]
SAMP8 mice (aging model)	Probiotic ProBiotic-4	2 × 10⁹ CFU/day by oral gavage; once daily for 12 weeks (aged SAMP8 mice).	Improved memory, reduced IL-6 and TNF-α, decreased microglial activation	[59]
In vitro / in silico	Myricetin (flavonoid)	In vitro: concentration‑dependent inhibition of SOD1 aggregation (IC₅₀ ≈ 15 µM).	Inhibited SOD1 aggregation	[49]

**Table 2:** Clinical studies on gut microbiota, diet, vitamins, and ALS

Population	Study focus	Main findings	References
25 ALS patients vs. 25 controls	Microbiota analysis	↓ *Firmicutes*, ↑ *Bacteroidetes*, reduced diversity	[28]
20 ALS patients vs. controls	Microbiota analysis	↓ *Firmicutes* and *Megamonas*, ↑ Bacteroidetes	[29]
66 ALS patients, 61 controls	Microbiota analysis	↓ *Eubacterium* rectale, *Roseburia* intestinalis	[33]
ALS patients vs. controls	Microbiota analysis	↓ *Succinivibrionaceae*, *Lachnospiraceae*; ↑ Streptococcaceae	[35]
57 ALS vs. 57 controls	Vitamin D levels	No significant differences, no correlation with ALSFRS-R	[60]
100 ALS patients	Vitamin D vs. survival	No survival effect; age and bulbar onset were key predictors	[61]
37 ALS patients	Vitamin D supplementation (2000 IU/day, 9 months)	↑ Vitamin D levels, slower ALSFRS-R decline after 9 months	[62]
>950,000 individuals (cohort)	Vitamin E intake	↓ ALS mortality risk (trend *p* = 0.004)	[63]
289 ALS patients	Vitamin E vs. placebo	No significant differences in progression	[64]
ALS patients (RCT)	FMT vs. placebo	Safe, favorable microbiota changes, slower disease progression	[7]
1 ALS patient (case report)	Washed microbiota transplantation	Improved motor function, relapse after interruption, improvement after resumption	[65]

ALS — amyotrophic lateral sclerosis; ALSFRS-R — Amyotrophic Lateral Sclerosis Functional Rating Scale–Revised; FMT — fecal microbiota transplantation; p — p-value; RCT — randomized controlled trial

↑ increase; ↓ decrease

### Vitamins support neuronal function and defense mechanisms against neurodegeneration in ALS

Vitamins play a key role in maintaining homeostasis in the body, including regulating the functioning of gut microbiota. By influencing the metabolism, bioavailability, and biological activity of many vitamins, gut microorganisms participate in both neuroprotective and neurodegenerative mechanisms [66]. Disorders of the gut microbiota composition may contribute to reduced vitamin absorption, but also exacerbate chronic inflammation, which is considered one of the main factors in the pathogenesis of ALS [66]. Among the vitamins most commonly studied in people with ALS, vitamin D holds a special role. Firstly, vitamin D affects both the regulation of the immune system by modulating the inflammatory response and the maintenance of blood-brain barrier integrity. Vitamin D has been also shown to modulate the composition and function of gut microbiota, which may have further consequences for neurological health [67]. Vitamin D deficiency can disrupt the immune homeostasis and promote inflammation. In a retrospective study conducted by Libonati et al. [60] vitamin D levels were assessed in 57 patients with ALS and 57 healthy volunteers in the control group. No statistically significant differences in vitamin D levels were found between both groups (mean 18.8 ± 12.2 ng/dl in the ALS group vs. 20.7 ± 10.1 ng/dl in the control group). Furthermore, vitamin D levels did not correlate with clinical parameters such as the Medical Research Council (MRC) scale, the revised ALS functional scale (ALSFRS-R), or vital capacity (FVC). In addition, vitamin D supplementation in some patients did not improve these parameters compared to the untreated group [60]. A study conducted by Yang et al. [61] assessed vitamin D levels in 100 patients with ALS and their association with bone mineral density and survival. Survival analysis using the log-rank test showed that survival rates for patients with vitamin D deficiency (< 10 ng/ml) and without deficiency (≥ 10 ng/ml) did not differ significantly. Cox regression analysis indicated that the most important prognostic factors were older age at symptom onset and bulbar onset, after taking into account other clinical factors [61].

In contrast, another study, which assessed vitamin D levels in ALS patients and the effect of supplementation on motor function, involved 37 ALS patients, with 81% having vitamin D levels below 30 ng/ml and 43% below 20 ng/ml. Twenty patients took 2000 IU of vitamin D daily for nine months. After six months of supplementation, vitamin D levels increased from a median of 18.5 ng/ml to 31.0 ng/ml [62]. After nine months, the decline on the ALSFRS-R functional scale was less profound in the vitamin D-supplemented group (*p* = 0.02), while no significant differences were observed after 3 and 6 months. This study shows that nine-month supplementation with vitamin D at a dose of 2000 IU per day is safe and may have a beneficial effect on motor function in patients with ALS. However, the authors emphasize the need for further studies to confirm these results [62]. It is not known whether vitamin D supplementation affects the course of ALS, as the results of previous studies are inconclusive. The relationship between vitamin D supplementation and the development or progression of ALS remains unclear and requires further research. The conflicting findings regarding vitamin D levels and supplementation in ALS may stem from several factors. Clinical heterogeneity among patients, such as differences in disease stage or progression rate, could influence outcomes. In addition, genetic variability affecting vitamin D metabolism, variability in dosing and duration of supplementation protocols, and differences in timing of intervention (e.g., early vs. late in the disease course) likely contribute to inconsistent results. These variables should be considered when interpreting the evidence, and highlight the need for more standardized, controlled studies [62].

Vitamin E, known for its strong antioxidant properties, is another vitamin of potential importance in ALS treatment [68]. Oxidative stress and free radicals are among the main factors damaging neurons in ALS [68]. In a prospective study involving 957,740 people aged 30 years and older, it was shown that long-term supplementation with vitamin E for at least ten years was associated with a significant reduction in the risk of death from ALS compared to people who did not use vitamin E (p for trend = 0.004) [63]. Although this results suggest a neuroprotective effect, data from intervention studies are inconclusive. Another study involving 289 patients with ALS, no significant differences in disease progression were observed between the α-tocopherol group and the placebo group [64]. These discrepancies may result from differences in the type of study (observational vs. interventional), dosing, duration of supplementation, and individual differences in the vitamin status and gut microbiota of participants. Epidemiological analyses have also evaluated the effects of other vitamins and compounds with antioxidant activity. For example, a study that analyzed data from over 1.1 million people, found that although vitamin C intake was not significantly correlated with the risk of developing ALS, higher intake of carotenoids, such as β-carotene and lutein, was associated with a significant reduction in the risk of developing the disease (RR = 0.75; 95% CI: 0.61–0.91) [69]. Carotenoids, as powerful antioxidants, may protect nerve cells from oxidative damage and modulate the inflammatory response. The action of vitamins in ALS is not limited to their direct effect on neurons. Their role in modulating the gut microbiota-brain axis is increasingly being emphasized, which may be crucial in the development and progression of neurodegenerative diseases. For example, vitamin B12, which is essential for the synthesis of myelin sheath and proper nerve conduction, can be synthesized by certain intestinal bacteria [70]. Its deficiency can impair neurological functions and exacerbate neurodegenerative processes. Vitamin A, in turn, supports the maturation of immune system cells and has antioxidant properties, which may protect neurons from damage. Vitamin K promotes the growth of beneficial gut bacteria that produce SCFAs. These compounds have anti-inflammatory effects and support the integrity of the intestinal barrier, which may indirectly modulate the immune response and influence the course of neurodegeneration. The accumulated scientific data indicate that vitamin deficiencies are common among ALS patients and may have a significant impact on the course of the disease. Although epidemiological studies suggest that supplementation with selected vitamins, especially those with antioxidant and immunomodulatory properties, may have a potential protective effect, the results of intervention studies remain often inconsistent and require further, better-designed clinical trials [70] This discrepancy can be explained by the complexity of ALS itself, individual differences in genetics and environment, as well as variability in the timing of supplementation, dosage, and the status of the patients’ gut microbiota [71].

### Probiotics support gut microbiome balance and mitigate neurodegenerative processes in ALS

Probiotics affect the gut microbiota and may have a significant impact on the course of neurodegenerative diseases. Alteration of the gut microbiota composition by these substances may affect inflammation, immune system function, and neuroinflammatory processes, which play a key role in ALS [72].

Probiotics are live microorganisms that, when consumed in adequate amounts, have a beneficial effect on the host’s health [73]. Probiotics modulate the gut microbiota, promoting the growth of bacteria with anti-inflammatory and neuroprotective effects can be of particular significance in ALS [74]. Probiotic bacteria, such as *Lactobacillus* and *Bifidobacterium*, support SCFAs production [74]. It was observed that the administration of oxalate to mice with ALS prolonged their survival by an average of 38 days [37]. These results suggest that oxalate, a natural metabolite produced by bacteria, can effectively support the restoration of microbial balance and intestinal function. In addition, butyrate therapy in mice with ALS led to a significant reduction in the number of abnormal Paneth cells in the intestine. Normalization of lysozyme 1 and defensin 5α levels was also observed, which improved intestinal structure and function, and slowed down pathological changes [37]. A study in rats evaluating the effect of B-GOS (Bimuno-galactooligosaccharides) prebiotic supplementation on cognitive function and neuroinflammation after surgery, conducted onseveral dozen adult rats that were given water with B-GOS or water alone for three weeks after surgery, revealed that B-GOS treatment improved efficacy of memory in the novel object recognition test compared to the control group [75]. Additionally, rats treated with B-GOS manifested reduced microglial activation and decreased expression of inflammatory markers such as iNOS, CD68, and IL-6. These results suggest that B-GOS may exert neuroprotective effect through the regulation of inflammation and modulation of the gut microbiome [75]. In another study [59], 30 SAMP8 mice aged nine months, treated with probiotic ProBiotic-4 (*Lactobacillus acidophilus*,* Bifidobacterium longum*,* Bifidobacterium bifidum*,* and Lactobacillus rhamnosus;* 2 × 10⁹ CFU per day) for twelve weeks, showed a significant memory improvement, assessed using Y-maze and passive avoidance tests. memory improvement. Additionally, the analysis of gut and brain microbiota composition in the same cohort of rats revealed that the probiotic restored microbial balance and reduced microglial activation. In the brains of mice treated with ProBiotic-4, a reduction in inflammatory cytokine levels, such as IL-6 and TNF-α, and a decrease in DNA damage markers, such as γ-H2AX were observed. The study also found inhibition of TLR4 receptor expression and the NF-κB pathway, indicating a reduction in inflammation and oxidative stress in the brain. The results suggest that probiotic supplementation may improve cognitive function in aging mice by modulating the microbiota-gut microbiota-brain axis and reducing neuroinflammation [59]. While these preclinical findings are promising, evidence from human studies remains limited. Further clinical research is required to verify whether similar probiotic effects occur in ALS patients.

It is still unknown which specific probiotic strains are most effective in the context of neurodegenerative diseases such as ALS. Differences in the composition of the patients’ gut microbiota suggest that probiotic therapy should be individualized. Further clinical studies with larger patient groups and longer follow-up periods are also needed. Another interesting direction is the combination of probiotics with a diet rich in prebiotics, which may enhance the beneficial effects of microorganisms. Currently, efforts are underway to develop so-called psychobiotics – strains targeting the gut microbiota-brain axis. Their potential use in ALS may bring new therapeutic opportunities in the future [72].

### Fecal microbiota transplantation promotes gut-neuronal balance and mitigates ALS progression

Fecal microbiota transplantation (FMT) is a procedure involving the transfer of functional bacterial flora from healthy donors to the gastrointestinal tract of patientsaiming to restore normal gut microbiota and treat both intestinal and extraintestinal disorders [76]. This method has gained recognition mainly in the treatment of recurrent *Clostridioides difficile* infections, but a growing number of studies indicate its potential in the treatment of neurological disorders, including autism spectrum disorders and ALS [77]. A case report revelaed a marked slowing of disease progression and improvement in motor function assessed by the ALSFRS-R scale in a 54-year-old female patient with ALS who underwent washed microbiota transplantation (WMT), an improved and safer variant of FMT [65]. After the injury requiring antibiotic treatment, the clinical condition of the patient deteriorated but improved rapidly after WMT was re-administered. This case highlights the potential of microbiota therapy in modulating the course of ALS [65]. Several recent clinical studies have investigated the role of gut microbiota in ALS patients, including both observational and interventional approaches [7, 65, 77, 78]. A multicenter study analyzing microbiota composition in ALS cohorts identified consistent patterns of dysbiosis, including reduced abundance of SCFA-producing bacteria and increased pro-inflammatory taxa, although inter-individual variability was high. Notably, Feng et al. [7] conducted a randomized, double-blind, placebo-controlled trial assessing the safety and efficacy of FMT in patients with sporadic ALS. The study demonstrated that FMT was safe, led to favorable changes in microbiota composition, and appeared to slow disease progression. While these findings are promising, limitations such as small sample sizes, short follow-up periods, and heterogeneity in patient populations must be taken into account. Collectively, available human studies suggest that microbiota modulation may hold therapeutic potential in ALS, but more rigorous, large-scale clinical trials are needed to establish causality and optimize intervention protocols [7].

## Conclusions and future perspectives

Growing evidence suggests that the gut microbiome plays an important role in the pathogenesis of ALS, influencing the nervous system via the gut microbiota-brain axis and modulating inflammation and immune response. Diet is an important factor in the course of ALS, affecting patients’ health by influencing both energy metabolism and the composition of gut microbiota. Weight loss, nutrient deficiencies, and metabolic disorders are common in this disease and may accelerate neurodegeneration.

Appropriate dietary intervention, especially a diet rich in antioxidants and anti-inflammatory ingredients such as polyphenols, may have a beneficial effect on the course of disease. Polyphenols have the ability to modulate the gut microbiota, increasing the proportion of bacteria that are beneficial to gut and nervous system health and limiting the growth of pro-inflammatory microorganisms, thereby supporting the production of metabolites with neuroprotective effects and reducing chronic inflammation, which is one of the key mechanisms of neuron damage in ALS. In addition, some bioactive compounds present in the diet, such as flavonoids, may directly influence pathogenic processes in nerve cells, e.g., by inhibition of the aggregation of proteins responsible for neuron degeneration. Furthermore, high-fat diets, including ketogenic diets, gain importance as potential metabolic support for neurons, offering an alternative energy source while influencing the composition of the microbiome.

While modulation of the gut microbiota represents a promising therapeutic strategy in ALS, significant challenges remain in translating preclinical findings into effective and safe clinical interventions. Firstly, most mechanistic insights have been derived from animal models, particularly SOD1-SOD1-G93A mice, which do not fully replicate the complexity of human ALS—especially the sALS form that constitutes over 90% of cases. Interventions that show neuroprotective effects in mice, such as specific probiotics or FMT, may not produce comparable outcomes in humans due to species-specific host–microbe interactions. Safety considerations also represent a major hurdle. Probiotics and FMT, although generally considered safe, carry risks such as infection, unintended immune activation, and potential transfer of antibiotic resistance genes. In immunocompromised ALS patients, even low-risk microbial interventions require careful risk–benefit assessment. Currently, there is a lack of long-term safety data for microbiome-targeted therapies in neurodegenerative conditions. Regulatory frameworks for microbiome-based interventions remain underdeveloped. In most countries, FMT is regulated as a drug or biologic, requiring rigorous clinical trial data and manufacturing under GMP (Good Manufacturing Practice) standards. Probiotics intended for therapeutic use may need to be registered as live biotherapeutic products (LBPs), which entails a higher regulatory threshold than food supplements. Finally, individual variability in microbiome composition poses a critical challenge to the standardization and personalization of treatment. Factors such as age, diet, genetics, medication use, and comorbidities influence both baseline microbial profiles and response to interventions. This underscores the need for stratified or personalized approaches rather than one-size-fits-all therapies. The development of predictive biomarkers and responder profiles may be essential for future clinical success. Alterations in the gut microbiota may influence ALS pathogenesis through several interrelated mechanisms involving the gut microbiota–brain axis. One key pathway is immune modulation: dysbiosis can lead to increased intestinal permeability (“leaky gut”), allowing microbial-derived molecules such as LPS to translocate into circulation. This can trigger systemic inflammation and microglial activation, both of which are implicated in motor neuron degeneration. Additionally, microbiota-derived SCFAs—such as butyrate and propionate—are known to regulate neuroinflammation and blood–brain barrier integrity; reduced SCFA production has been reported in ALS patients and animal models. Another crucial mechanism involves microbial metabolism of tryptophan and nicotinamide, which affects CNS function via modulation of the aryl hydrocarbon receptor (AhR) and sirtuin signaling. For example, *Akkermansia muciniphila* has been shown to increase nicotinamide levels, which promote neuronal survival in ALS mouse models. Moreover, microbiota can modulate oxidative stress by altering host antioxidant capacity and glutathione metabolism. Dysbiosis may also influence mitochondrial function, a known factor in ALS progression, through the production of bioactive metabolites and signaling lipids. Lastly, the gut microbiota may impact neuromuscular communication directly via microbial production of neuromodulators such as GABA, serotonin, and dopamine precursors, which interact with the enteric and central nervous systems. These mechanisms suggest that microbiota changes are not merely epiphenomena, but may actively contribute to ALS progression, especially when interacting with genetic and environmental risk factors. ALS is a heterogeneous disease with diverse genetic and clinical presentations, which may influence the composition of gut microbiota and affect individual responses to microbiota-targeted therapies, highlighting the need for personalized approaches in future research and clinical applications. Given the clinical heterogeneity of ALS, including differences in disease progression, metabolic status, and nutritional needs, a one-size-fits-all dietary approach may be insufficient. Additionally, genetic factors and individual variations in gut microbiota composition may modulate the response to specific nutrients or interventions. Therefore, future research should focus on developing personalized nutrition strategies that take into account patients’ clinical profiles, genetic backgrounds, and microbiota characteristics to improve therapeutic outcomes and quality of life.

In the future, the development of nutritional therapies targeting the modulation of microbiome and cellular metabolism in ALS may be a promising direction for research. A personalized approach to diet, based on the analysis of individual microbiome and metabolic profiles of patients, may optimize nutritional support and slow disease progression. The integration of specific bioactive supplements, such as polyphenols or flavonoids, along with monitoring of clinical effects, may also contribute to the improvement of the quality of life of ALS patients. Further clinical and experimental studies are needed to precisely determine the effectiveness of such interventions and to develop nutritional guidelines for this disease.

To support clinical decision-making and future research prioritization, microbiota-targeted interventions in ALS should be assessed according to the strength of evidence, feasibility, safety, and translational readiness. Current data provide increasing evidence for both robustness and applicability of such interventions. Probiotics represent the most clinically accessible intervention, with several small-scale human studies and preclinical models suggesting potential anti-inflammatory and neuroprotective effects; however, strain-specific effects, dosing regimens, and long-term efficacy remain insufficiently understood. Dietary interventions, such as high-fiber, Mediterranean-style, or ketogenic diets, are supported by indirect evidence from observational studies and animal models; these approaches are feasible and low-risk but lack randomized controlled trials specific to ALS. Vitamin supplementation, including vitamins D, E, B12, and nicotinamide, has shown mixed results in human studies; while mechanistically plausible, current data are insufficient to support widespread use beyond addressing deficiencies. FMT remains in the experimental stage, with promising effects observed in mouse models but virtually absent human data; the procedure carries greater regulatory and safety challenges. Based on current evidence, probiotic supplementation and dietary modulation appear to be the most feasible and justifiable interventions for near-term clinical exploration, whereas FMT and microbial metabolite targeting require further mechanistic validation before translation.

## Study limitations

A major limitation of research investigating the gut microbiota in ALS lies in the considerable methodological heterogeneity of the studies, which significantly impacts the reliability and comparability of findings. Most clinical studies are conducted on small cohorts - often fewer than 36 participants - limiting their statistical power and increasing the risk of type I and type II errors. The lack of longitudinal follow-up in many of these studies further constrains our understanding of temporal changes in microbiota composition in relation to disease progression. Moreover, numerous studies fail to properly control for critical confounders such as diet, antibiotic usage, BMI, metabolic status, and the presence of gastrointestinal symptoms—each of which can independently influence microbiota composition.

In terms of microbiome analysis, there is also a lack of standardization in sample collection (e.g., stool vs. mucosal swabs), DNA extraction protocols, 16 S rRNA sequencing regions targeted (e.g., V1–V3 vs. V4–V5), and bioinformatics pipelines. Such inconsistencies hinder cross-study comparisons and meta-analytic efforts. Additionally, most studies rely solely on 16 S rRNA sequencing, which provides limited taxonomic and functional resolution compared to shotgun metagenomics or metabolomics. Functional insights into the microbial metabolites and host–microbe interactions are therefore often speculative or missing entirely.

Another significant methodological challenge is the frequent absence of proper control groups - either healthy individuals or patients with other neurodegenerative disorders - to assess the specificity of microbiota changes in ALS. Some studies also exhibit publication bias by selectively reporting only statistically significant differences without discussing negative or null results. Lastly, few trials investigating interventions (e.g., probiotics, FMT, or diet) use randomization, blinding, or placebo control, which raises concerns regarding internal validity and reproducibility of therapeutic effects.

## Data Availability

No datasets were generated or analysed during the current study.

## Abbreviations

AhR: Aryl hydrocarbon receptor
ALS: Amyotrophic lateral sclerosis
B: GOS-Bimuno-galactooligosaccharides
BMI: Body mass index
CNS: Ccentral nervous system
fALS: Familial amyotrophic lateral sclerosis
FMT: Fecal microbiota transplantation
HPA axis: Hypothalamic-pituitary-adrenal axis
HFD: High-fat diet
GABA: γ-aminobutyric acid
GMP: Good manufacturing practice
IL: 6-interleukin-6
LDL: Low-density lipoprotein
LBP: Live biotherapeutic products
LPS: Lipopolysaccharide
MCT: Medium-chain triglyceride
SCFA: Short-chain fatty acid
sALS: Sporadic amyotrophic lateral sclerosis
TLR: Toll-like receptor
TNF: α-tumor necrosis factor-α
WMT: Washed microbiota transplantation
